# Medical Opinion and Movement

**Published:** 1908-04-25

**Authors:** 


### Medical Opinion and Movement.

THE ætiology of appendicitis and the increased incidence of the disease in recent years are still questions for discussion and consideration. Some light may be thrown upon the problem by a comparative study of the incidence of the disease in different countries and among different nations. Recent reports from China and India testify to the extreme rarity of the disease in these two countries. According to the accepted views as to the causation of the disease, the Chinese should not be so free from this trouble. Dr. John MacGowan, of Amoy, writes that in the opinion of foreign physicians who treat large numbers of natives in hospitals, a large majority of the population are below par, and suffer from indigestion, and Mr. D. Christie, of Mukden, says: "If dyspepsia, indigestible food, and bolting have any share in the causation of appendicitis, we should have plenty of it here." It is suggested that the absence of the disease may be explained by differences in the anatomical relationships of the appendix. The ban by the Government on human dissection and post-mortem examinations makes research in this direction impossible.

MR. JAMES HARRIS has attempted to deal with the question in relation to the natives of India. It has been suggested that the simple diet of the people may account for their immunity from appendicitis. The absence of violent exercise has also been put forward as a possible influence. Mr. Harris, however, rejects these explanations. It will be remembered, too, that Mr. W. H. Battle has suggested that the introduction of steel rollers for flour milling may be responsible for the increase of the disease in America and Great Britain, as steel particles can be found in the flour, and it has been pointed out that steel ground flour is unknown in India. Mr. Harris, however, contends that in place of the steel particles the grinding stones in India leave many stony particles in the ground material, which should be equally capable of setting up the disease. He offers a two-fold explanation. He suggests in the first place that the habitual and excessive purgation which occurs in India prevents the lingering of irritating materials in the cæcum, and he refers to the natural squatting attitude adopted by the natives in defæcation as a second possible factor in the prevention of the disease. By this attitude the cæcum is well compressed and thoroughly emptied of its contents. These considerations at any rate suggest that a further investigation into the habits and customs of such people as are comparatively free from the disease may throw further light upon the ætiology of the disease and the cause of its frequency among the more civilised nations.

CALMETTE'S ophthalmic reaction for tuberculosis has already been discussed and commented upon in these columns on more than one occasion. Although it is less than a year ago when it was first introduced to the profession, it has received considerable attention in all parts of the world. The reports of many observers after extensive trials have on the whole confirmed the diagnostic value of the reaction originally claimed for it by Professor Calmette. It must be recognised however, that, like every other reaction of a similar nature, it is not absolute. It may fail in cases in which other considerations definitely point to the presence of a tuberculous infection, and positive results are recorded in non-tuberculous cases. Some observers appear to obtain more satisfactory results than others. The innocuous nature of the reaction has also, on the whole, been borne out by more extended experience, although from time to time untoward results, in the nature of a severe conconjunctivitis have been recorded. Dr. Kremer of Vienna, on the other hand, has made a series of comparative observations to ascertain the relative value of Calmette's ophthalmic and Pirquet's cutaneous reaction. Without giving all the detailed figures of these observations, it may be said that on the whole Dr. Kremer considers that of the two methods Pirquet's cutaneous one is the more trustworthy, and at the same time it is easier, less severe, and less painful. If further observations confirm the greater trustworthiness of the cutaneous reaction, there can be no doubt that on other grounds it is preferable both for the physician and for the patient.

THE transmission of the diphtheria virus from the lower animals to man is still a question upon which opinion is divided. Clinically the disease observed among animals is identical in its characteristics with human diphtheria. It occurs among horses and cattle, it is exceedingly common among pigeons, and at times these birds are decimated by the disease. It is also common among other domestic birds—fowls, ducks, pheasants, parrots, etc. Bacteriologically, however, the causal organism has been held to be different, although several observers have isolated the bacillus diphtheriæ or a bacillus closely similar to it from cases of animal diphtheria. Dr. L. W. Sambon has recently reviewed the evidence in favour of an identity in the causal virus for human and animal diphtheria, and urges many arguments in regard to the origin, distribution, and spread of epidemics of the disease in support of the view that domesticated animals, and especially birds, play an important *rôle* in the epidemiology of human diphtheria. He points out that of all known bacteria, the bacillus diphtheriæ is one which exhibits the greatest variations. The recognition of differences between the causal microbe of diphtheria in man and other animals does not militate against the possibility of transmission of the disease from one to the other. Amongst other interesting facts bearing on the question he points out that in the French army for the period 1872-85 the relative proportion of diphtheria among cavalry and infantry was in the ratio of 10 to 3. In the German army, for a similar period, the proportion was 3 to 1. Dr.

Sambon is of opinion that birds, especially pigeons, are largely instrumental in the spread of the disease, and he makes the interesting suggestion that the plague which fell upon the people from eating quails, as described in Numbers xi. 31-34, was actually an epidemic of diphtheria. The whole question undoubtedly calls for further research and investigation, and, as Dr. Sambon suggests, the veterinary surgeon of the modern school should take his full share of work in such an inquiry.

---

UNTIL recent years it has generally been held that typhoid bacilli, in ordinary cases of typhoid fever, are confined to the bowel, the mesenteric gland, and the spleen. There is, however, a considerable volume of evidence accumulating to show that, even in ordinary cases, the bacilli are present throughout the body from comparatively early in the illness; in other words, that typhoid fever is not merely a toxæmia, but actually a septicæmia. The authority for this belief is based upon the published works of such men as Widal himself, Lesieur, and Courmont, Klinger, Conradi, Gennari, and many others. An attempt has recently been made to find the time-limits for this blood infection with typhoid bacilli; and it is particularly interesting, in regard to typhoid "carriers," to find that typhoid bacilli may still be cultivated from the blood of patients who have long since thrown off all the clinical signs of their active illness. The method employed has been to receive five cubic centimetres of the patient's blood into 300 cubic centimetres of bouillon, and then to deal suitably with the colonies of organisms that appear in the latter after incubation.

---

LESIEUR and Courmont had, until recently, failed to find typhoid bacilli in the blood before the fifth day of the declared disease; but Conradi showed, in one case, that they might be found there even during the period of incubation. The duration of the septicæmia has for some time been known to be considerable. Gennari, for example, found eight undoubted cases in which positive cultivations were given by the blood for periods varying from four to forty days after the end of the pyrexia. Lesieur has recently investigated thirty-three consecutive cases far into convalescence. The blood was taken from four to six weeks after apyrexia had become fully re-established, at the time when the patients, being fully restored to health, were about to return to their daily occupations. All cases of relapse and all cases in which there were complications were excluded. The results were that six out of the thirty-three patients still gave pure cultures of typhoid bacilli from their blood. The nature of the organism was tested in every known way, and it conformed with the requirements of the typhoid bacillus morphologically, culturally, in staining reaction, and by clumping. It follows therefore that after convalescence from typhoid fever the typhoid bacillus can persist in the blood, and be recovered therefrom, in 18 per cent. of patients for as long as from four to six weeks after all fever has gone. This is a very remarkable fact, and its bearing upon the questions of prolonged convalescence, sequelæ, and of typhoid "carrying" are so obvious that they need no further comment.

THE question of obtaining a mercurial cream for injection purposes, in which the percentage of mercury may be as high as possible, has already been referred to in these columns. The preparation then described was stronger than the well-known mercurial creams devised by Colonel Lambkin and Dr. Julius Althaus; its strength was such that 100 parts of the cream contained 20 parts by weight of mercury. M. Dumesnil has devised a still more concentrated preparation, one which, indeed, contains 40 parts, instead of 20 parts, of mercury per 100. The formula is as follows: Mercury, 40 grammes; sterilised wool fat, 26 grammes; sterilised liquid paraffin sufficient to produce 100 c.c. The details of the preparation of M. Dumesnil's cream have appeared in the *Pharmaceutical Journal*. The mercurial cream or grey oil thus obtained is fluid and stable at temperatures between 15° C. and 20° C. It is of a dark colour, rather black than grey. When carefully prepared the mercury is in an extremely fine state of subdivision; under the microscope the particles are smaller than starch grains, and they have been compared in size to the amorphous granules of a deposit of urates in a concentrated urine. There are as yet no records of the results obtained by the use of this particular form of mercurial cream in this country. The formula has been adopted by the Société de Pharmacie of Paris. It it chiefly in difficult cases of syphilis that it is likely to be tried. The purpose of the present note is to draw attention to the fact that this concentrated cream may be prescribed or dispensed, and used. It is possible that the local reaction at the site of inoculation may be disadvantageous; but, on the other hand, if the local reaction proves not to be any more severe than it is with the other forms of mercurial creams it will be a great advantage to be able to introduce a given quantity of mercury in a smaller bulk of material.

---

KOLZENBURG, who is one of Professor Kummel's assistants at the magnificent surgical clinic attached to the Hamburg-Eppendorf general infirmary, has just published an interesting *résumé* of the treatment of cases of general purulent peritonitis by operation. As the author remarks, in prefacing his list of cases treated at the general infirmary during the last few years, this condition of purulent peritonitis is one of the most difficult that comes under the care of the surgeon. At present the operative treatment is recognised as holding out the only chance of successful recovery. It is true there are cases on record of patients with "diffuse general peritonitis" who have recovered without operation, but none of these alleged recoveries are absolutely established, and grave doubts as to the diagnosis having been a correct one must remain in view of the fact that it is impossible to be absolutely certain that the case is one of "diffuse purulent peritonitis" unless the peritoneum has been opened and inspected at the operation or subsequently at the autopsy. The best established cases on record, where, from the symptoms and course it is reasonable to think that the case was really one of peritonitis, are probably cases of large localised inflammations or abscesses.

THE operative treatment of diffuse purulent peritonitis has not, however, been attended with a very marked decrease in the mortality. It is true the mortality has decreased, and decreased in a very encouraging fashion, since the more modern methods of treatment have won recognition; but it is still high, and surgeons are seeking, by various methods and in various ways, to perfect the technique of the operation and to promote recovery and shorten convalescence by careful after-management. In no two countries is the method of treatment of such cases absolutely the same, and the practitioner who is interested in comparative surgery cannot do better than study the methods used in the treatment of such cases here in England and abroad. Even in England different surgeons have different methods, and the after treatment is different in various hospitals. It is therefore interesting to see what is being done at the largest surgical clinic on the Continent, whereat yearly a large number of cases of general diffuse peritonitis (certainly a much larger number than occur yearly at any London hospital) are treated.

TO a large extent the method at Eppendorf is that which is followed at Heidelberg. The abdomen is opened: if the case is one of perforated appendix the appendix is rapidly removed. The abdominal cavity is flushed out with hot normal saline, no eventration being allowed, but the intestines "are thoroughly washed and adhesions between them are, wherever possible, broken down." All this is done very rapidly, and the wound is sewn up, only a small hole being left at the bottom of the incision for drainage. Through this a glass Dreesman's drain, containing a vioform gauze tampon, is passed, and the patient is put back to bed. The head of the bed is raised, but the patient is not placed in the Fowler position. The vioform tampon is changed daily, without disturbing the glass drain, which is removed four days after the operation and replaced by a much smaller one. Under this treatment, taking the figures for last year only, the mortality is only 9 per cent. Under the old method, in which counter-incisions were made and a much more vigorous treatment instituted, the mortality was between 40 per cent. and 50 per cent. These figures speak for themselves, and the results obtained at Eppendorf are remarkable enough to deserve the close attention of English surgeons.

SCHROTTER has examined the skin and subcutaneous tissues in cases of exophthalmic goiture, and the results of his investigations were communicated at a recent meeting of the Berlin Clinical Society. He found that there was marked fatty change in the skin, leading, in fact, to a condition of well-marked subcutaneous lipomatosis. The chemical analysis of this fatty subcutaneous tissue was carried out by Professor Pauzer, who found that it differed in many ways from normal subcutaneous tissue fat. Its melting-point was much higher than that of normal fat, and it was entirely deficient in cholesterin and lecithin. The author does not consider his results of much clinical value, but he strongly pleads for further research in this subject. The skin changes in this disease are very well known, but they have not as yet received the attention they deserve; and in view of the obscurity of the causation of the disease, any points which may throw light upon its ætiology or pathology must of necessity be welcomed.

LEINER and Knöpfelmacher, in an article dealing with the treatment of Dermatitis exfoliativa, declare that this condition can no longer be considered as a distinctive clinical entity, but must be accepted as a severe form of pemphigus neonatorum. Thus it has been known to occur in an epidemic of the latter disease, and has given rise to pemphigus directly by contagion. Langstein has recorded a case in which a midwife carried the infection from one child to another. The ætiology of the condition, however, is as yet quite obscure. The authors are in favour of mild measures in the treatment. All that is necessary is to protect the parts with an aseptic powder, and to give a daily tannic-acid bath (tannic acid 1 part, water 20 parts). After the parts have been well bathed with this lotion they are carefully dried and powdered. Constitutional treatment is at the same time undertaken. The authors have been very successful with their cases treated in this manner, their mortality being only 32 per cent. as against the 50 per cent. under the Prague method of treatment.

IT is a familiar fact that solutions of adrenine have a tendency to become coloured on keeping. Messrs. Gunn and Harrison lately read an interesting paper upon this subject before the Pharmaceutical Society in London, and incorporated in their conclusions the results of some important pharmacological tests conducted by Professor W. E. Dixon, of King's College. Prominent factors in the discoloration which occurs seem to be the exposure of the solution to air, and its contact with alkaline glass; and, to a less degree, exposure to light and the presence of minute traces of ferric salts. The authors have found that a solution which does not readily discolour, even when exposed to light and air for several weeks, can be obtained by using 0.3 part of real hydrochloric acid to each part of adrenine—*i.e.* about half as much again as one molecular equivalent. Professor Dixon's preliminary tests are of considerable importance. He finds that this discoloration is associated with greatly reduced physiological activity, and that the depth of the colour change is approximately proportional to the degree of diminished efficiency. Deeply coloured solutions over six months old may be practically inert. Professor Dixon also found that a fresh solution of natural adrenine was about twice as active as a fresh solution of synthetic adrenine prepared by the same firm of manufacturers.

